# An Account of the Good Effects of Fixed Air in a Case of Putrid Fever; and of a Profuse Hæmorrhage from the Ears Terminated by a Fit of Epilepsy

**Published:** 1783

**Authors:** 


III. *An account of the good effects of Fixed air in a caſe of putrid fever; and of a profuſe hæmorrhage from the ears terminated by a fit of epilepſy. By* F. J. Janſſens, *M. D. Phyſician at Ooſterhout; communicated in a letter to his Excellency Prince* Gallitzin, *the Ruſſian ambaſſador at the Hague, and by him to Dr.* Simmons, *through the hands of Mr.* J. H. de Magellan, F. R. S.

ON the 23d of Nov. 1779, I was called to Terheyder, near Breda, to conſult with Dr. Zegers, a phyſician of conſiderable reputation at the latter of thoſe places, on the caſe of a young man man of the name of Rieboom, whom he had attended eight or nine days. The patient was in his 26th or 27th year, and, when in health, of a robuſt conſtitution and florid complexion. He had been attacked about five weeks before with a putrid fever, in the marquiſate of Bergenop-zoom, from whence he had been removed to his father's; and when we ſaw him the ſymptoms were ſuch as threatened a ſpeedy and fatal termination. His pulſe was extremely quick, attended with vomiting, and delirium. From the 4th and 5th day of the diſeaſe he had been ſubject to hæmorrhages from the noſe, which ſtill continued. His countenance was now bloated, pale, and cadaverous; his breath very offenſive; the pupils of his eyes extremely dilated, and he fainted away on the leaſt motion.—We preſcribed the liberal uſe of a ſtrong decoction of Peruvian bark with elixir of vitriol, and directed compreſſes dipped in a ſolution of white vitriol to be applied to the noſtrils. This method was continued, but without any viſible good effect, till the 25th of November, when we repeated our viſit. I then recollected the Engliſh method of adminiſtering fixed air in clyſters, which your Excellency did me the honour to communicate to me, and having mentioned it to my colleague we agreed to

 give give it a trial, though, I confefs, without any hopes of fuccefs. A large bladder full of fixed air was directed to be injected every four hours, and within a few hours after this practice had been adopted the hæmorrhage at the nofe ceafed. By perfevering in it for a few days the fymptoms of putridity and fever gradually abated, and the patient in a fhort time recovered.

The other cafe which I take the liberty of communicating to your Excellency, is that of a young woman, 25 years of age, fervant to an innkeeper at Oofterhout, named Vandewygert. She had complained for feven weeks of ftomach complaints, head-ach, and other fymptoms that feemed to be occafioned by obftructed menfes, when, on the 20th of November 1782, fhe was feized with a profufe hæmorrhage from both ears, which continued without intermiffion tho' not always with the fame violence, till the 1ft of December, and when, as fometimes happened, the cavities of the ears were filled with *coagula*, the blood flowed from the mouth and noftrils. By this conftant drain of the vital fluid, the powers of life were fo exhaufted, that her pulfe was hardly to be felt, and her countenance was truly cadaverous. In this reduced ftate fhe was attacked with a fit of epilepfy, of uncommon violence, violence, which continued for the ſpace of ſixty hours. Before ſhe had been two hours in this fit the hæmorrhage ſtopped, and never returned afterwards. On the 8th of December the menſes re-appeared, and ſhe is now perfectly recovered.

*Ooſterhout,*

*Jan.* 10, 1783.

